# Low IL-6, IL-10, and TNF-*α* and High IL-13 Cytokine Levels Are Associated with Severe Hepatic Fibrosis in* Schistosoma mansoni* Chronically Exposed Individuals

**DOI:** 10.1155/2018/9754060

**Published:** 2018-01-23

**Authors:** Mable M. Mutengo, Takafira Mduluza, Paul Kelly, James C. L. Mwansa, Geoffrey Kwenda, Patrick Musonda, James Chipeta

**Affiliations:** ^1^Department of Pathology and Microbiology, University Teaching Hospital, Lusaka, Zambia; ^2^University of Zambia, Lusaka, Zambia; ^3^Department of Biochemistry, University of Zimbabwe, Mount Pleasant, Harare, Zimbabwe; ^4^Department of Internal Medicine, School of Medicine, University of Zambia, Lusaka, Zambia; ^5^Department of Biomedical Sciences, School of Health Sciences, University of Zambia, Lusaka, Zambia; ^6^Department of Epidemiology and Biostatistics, School of Public Health, University of Zambia, Lusaka, Zambia; ^7^Department of Pediatrics and Child Health, School of Medicine, University of Zambia, Lusaka, Zambia

## Abstract

Several studies have attributed the etiopathogenesis of chronic* Schistosoma mansoni* related hepatic fibrosis to unregulated immune responses against trapped parasite ova in the host. However, there is limited data on immune profiles associated with varying degrees of the disease in a population under chronic exposure to the parasite. We therefore investigated the role of selected T-helper (Th)1, Th2, and Th17 cytokines in relation to hepatic fibrosis severity among individuals resident in a hyper-*Schistosoma mansoni* endemic region of Western Zambia. Two hundred and forty-four* S. mansoni* infected individuals with and without fibrosis were analysed for cytokine profiles. Based on hepatic fibrosis stage as determined by ultrasound, participants were categorized into Group 0, Group I, Group II, and Group III. Cytokines were measured in* S. mansoni* egg stimulated whole blood culture supernatants using the BD Cytometric Bead Array kits. Compared to the nonfibrotic group, participants in the severe hepatic fibrotic group produced less interleukin- (IL-) 6, IL-10, and tumour necrosis factor-alpha (TNF-*α*). On the other hand, IL-13 was significantly elevated in this group compared to the nonfibrotic group (*p* < 0.001). Our results suggest that low IL-6, IL-10, and TNF-*α* and high IL-13 levels may influence* S. mansoni* disease progression.

## 1. Introduction

In endemic areas,* Schistosoma mansoni* disease presentations are often subtle and unrecognized. However, in a subset of infected individuals, severe hepatic fibrosis occurs due to excessive deposition of extracellular matrices in portal spaces in response to trapped* Schistosoma* egg leading to presinusoidal fibrosis and portal hypertension [1, 2]. In such individuals, complications such as splenomegaly, ascites, and esophageal varices that lead to life threatening haemorrhage may develop [1].

The pathology observed in* S. mansoni* disease is attributed to unregulated CD4^+^-T cell immune responses directed against trapped parasite ova in the hepatic sinusoids [3]. Studies have demonstrated that CD4^+^T-helper (Th)1 and Th2 immune responses are responsible for the immunopathology observed in acute and chronic schistosomiasis [3, 4], respectively. As egg deposition builds up, Th1 immune responses are dampened and a more pronounced Th2 response is observed [5]. This process is characterized by a granulomatous reaction with subsequent fibrosis [6], which is dependent mainly on Th2 mediated cytokines including interleukin- (IL-) 4, IL-5, and IL-13 [7–9]. Among the Th2 cytokines involved in the pathogenesis of* S. mansoni*, IL-13 has been identified as a key profibrotic cytokine in the development of* Schistosoma* induced hepatic fibrosis, the main cause of morbidity and mortality in chronic* S. mansoni* disease [8–10].

While it has been previously reported that Th1 immune responses are associated with acute infection [5], studies are now suggesting that Th1 cytokines such as IFN-*γ* and TNF-*α* could be involved in regulating hepatic fibrosis observed in chronic schistosomiasis disease. IFN-*γ* is a known antifibrogenic cytokine [11] that has been shown to confer protection against fibrosis in* S. mansoni* infected individuals [12, 13]. IFN-*γ* inhibits the synthesis of extracellular molecules by the hepatic stellate cells [11, 14] and enhances matrix metalloprotease (MMP) gene expression [15]. Low levels of this cytokine are reported to be associated with severe periportal fibrosis in* S. mansoni* infected people [2]. On the other hand the role of tumour necrosis factor-alpha (TNF-*α*) in regulating schistosomiasis disease remains unclear. Whereas some studies have documented that TNF-*α* is protective against severe disease [16], high levels are shown to aggravate disease [12, 17, 18]. Recently, IL-17A, a Th17 cytokine, was shown to contribute to the immunopathogenesis of schistosomiasis in both human and experimental animal models [19, 20], perhaps by inducing the deposition of cellular and collagen substances in the liver [21]. However, data on whether IL-17A is associated with severe* S. mansoni* hepatic disease in humans is limited. Furthermore, the role of Th1 and Th2 cytokines in different stages of* S. mansoni* disease in a population persistently exposed to* Schistosome* parasites remains understudied.

We have previously described hyperendemic foci of* S. mansoni* infection in Western Zambia [22]. In the current paper, we report findings on the role of selected Th1, Th2, Th17, and regulatory cytokines in individuals with varying stages of hepatic fibrosis in the same population.

## 2. Materials and Methods

### 2.1. Study Area and Population

The study population was randomly drawn from a* S. mansoni* hyperendemic region of Kaoma District in the Western province of Zambia as reported in detail elsewhere [23]. Participants positive for* S. mansoni* infection as determined by Kato-Katz or serology were recruited. Two hundred and fifty-five participants with varying stages of hepatic fibrosis were recruited into the study [23]. Eleven of the recruited participants were excluded from analysis as they did not meet the inclusion criteria. Due to limited community social infrastructure such as clean water supply and sanitation, residents are repeatedly exposed to* S. mansoni* parasites as they conduct their daily activities in the infested rivers. This study was approved by the University of Zambia Biomedical Research Ethics Committee (IRB00001131).

Based on ultrasound liver image pattern findings,* S. mansoni* infected participants were categorized into four groups: Group 0, infected with no fibrosis (*n* = 111); Group I, infected with mild fibrosis (*n* = 59); Group II, infected with moderate fibrosis (*n* = 43); and Group III, infected with severe fibrosis (*n* = 31). Prior to participation into the study, written informed consent and/or assent, where appropriate, was sought from potential participants. Participants with schistosomiasis were treated with 40 mg/kg body weight praziquantel while those with associated severe disease were referred to the nearest health facility for further management.

### 2.2. Parasitology and Serology for* S. mansoni* Infection

Stool samples from recruited participants were examined for the presence of* S. mansoni* infection by the Kato-Katz method as described previously [22]. Intensity of infection was classified based on the World Health Organization (WHO) criterion as light (1–99 epg), moderate (100–399 epg), and heavy (≥400 epg) [24]. A serological Enzyme linked immunosorbent assay (ELISA) test for* Schistosoma* antibodies (Scimedx Corporation, Denville, NJ 07834, USA) was used to confirm exposure in participants with negative Kato-Katz readings. Absorbance of serum samples conjugated with* Schistosoma* antibodies was read on an EL-800 ELISA plate reader (BioTek Instruments Inc) and results were reported according to the kit manufacturer's instructions.

### 2.3. Ultrasound Evaluation for* S. mansoni* Related Morbidity

*S. mansoni* related hepatic morbidity was detected using a portable ultrasound machine (Mindray DP-1100 Plus electronic convex transducer: 35C50EB, 2.5/3.5/5.0 MHz, Shenzhen Mindray Bio-Medical Electronics Co., Ltd, Nanshan, Shenzhen 518057, China). Examinations were conducted by two ultrasound technicians, one of them having been trained in the detection of schistosomiasis-related morbidity under the Schistosomiasis Control Initiative Programme (SCI). The degree of hepatic fibrosis was defined based on the liver image patterns (IP) ranging from A to F [25]. Since these scores denote the degree of fibrosis, we therefore classified our participants into four groups as normal (A and B), mild fibrosis (C), moderate fibrosis (D), and severe fibrosis (E and F). Participants with IP unrelated to schistosomiasis graded X (fatty liver), Y (cirrhosis), and Z (undetermined) were excluded from analysis.

### 2.4. Stimulation of Whole Blood and Cytokine Analysis

Five milliliters of venous blood was collected from study participants into heparin vacutainers. The blood was diluted 1 : 4 with RPMI culture containing 2 mm L-glutamine, 100 u/ml penicillin, and 100 *μ*g streptomycin (Lonza Group, Ltd). The diluted blood was then transferred into 24-well culture plates containing 10 *μ*g/ml* Schistosoma* soluble egg antigen (SEA) (Theodor Bilharz Institute, Giza, Egypt) and was incubated at 37°C in the presence of 5% carbon dioxide for 24 hours. The culture supernatants were harvested into cryovials and stored initially at −20°C and later transported on ice to the research laboratory in Lusaka, Zambia, where they were stored at −80°C until required for analysis. To measure cytokine levels, the Human Th1/Th2/Th17 cytometric bead array (CBA) and the human IL-13 flex kits (BD Biosciences, San Jose, California) were used. The Th1/Th2/Th17 kit simultaneously detects IL-2, IL-4, IL-6, IL-10, TNF-*α*, IFN-*γ*, and IL-17A cytokines in a single sample whereas the IL-13 flex kit detects IL-13. The assays were performed according to the manufacturer's instructions. Samples and standards were acquired on the BD FACsverse cytometer (BD Biosciences, San Jose, California) and the generated FSC files were analysed using FCAP Array version 3.0 software.

### 2.5. Statistical Analysis

The Shapiro-Wilkinson test was used to test for normality of the cytokine data. We used the Mann–Whitney test to determine differences in cytokine levels between the two groups and the Kruskal-Wallis test for differences among the four groups. Differences in cytokine measurements between the nonfibrotic group (Group 0) and each of the 3 groups (groups I, II, and III) were determined by Dunn's test for multiple comparisons. We used ANOVA to test for differences in continuous demographic variables while, for categorical variables, the chi-square test or the Fisher's exact test was used where appropriate. All statistical tests were two-sided and *p* values < 0.05 were considered significant. Statistical analysis was performed using Stata version 12 software (STATA Corporation; College Station, TX) and Graphpad prism 6 software (GraphPad Software, San Diego, USA).

## 3. Results

### 3.1. Characteristics of the Study Participants

The overall median age in this study population was 34 years (IQR = 19–42). As shown in Table 1, the median age difference across the four groups varied significantly (*p* < 0.001). Although there were more female than male participants, the difference between the groups was not significant (*p* = 0.38). In this study, no significant differences in intensity of infection were observed across the four groups (*p* = 0.43).

### 3.2. Cytokine Levels in the Different Stages of Portal Fibrosis

Cytokine levels in whole blood culture supernatants of 244 participants were determined in relation to fibrosis (Table 2). Median IL-6, IL-10, and TNF-*α* cytokine levels were significantly elevated in participants without fibrosis than those with fibrosis (Table 2).

**Figure 1:**
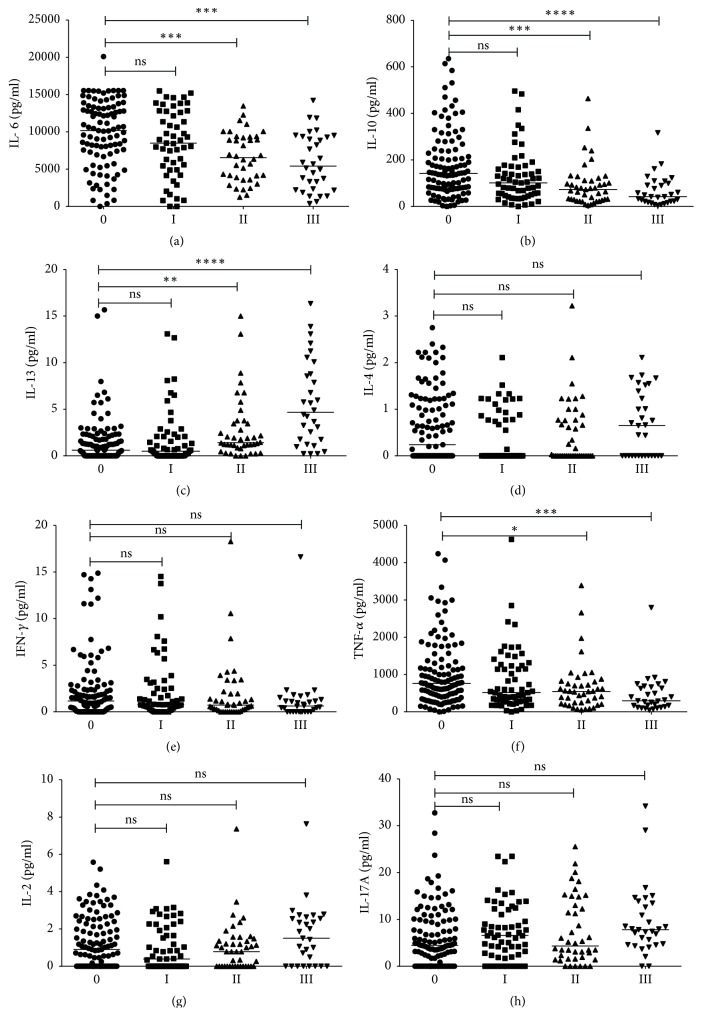
Cytokine levels in relation to degree of fibrosis. Median differences between Group 0 (control) and each of the three groups were determined using Dunn's test for multiple comparisons. Group 0 (infected with no fibrosis), Group I (infected with mild fibrosis), Group II (infected with moderate fibrosis), and Group III (infected with severe fibrosis). The significant level was set at ≤ 0.05 for a two-sided test. ^*∗*^*p* ≤ 0.05, ^*∗∗*^*p* ≤ 0.01, ^*∗∗∗*^*p* ≤ 0.001, and ^*∗∗∗∗*^*p* ≤ 0.0001.

On the other hand individuals with fibrosis produced more IL-13 than those without fibrosis. No statistical significance in IL-2, IL-4, IFN-*γ*, and IL-17A was observed between the two groups. A similar trend was observed when cytokines were analysed based on the degree of fibrosis (Figure 1). Individuals in Group II and Group III produced less IL-6, IL-10, and TNF-*α* (Figures 1(a), 1(b), and 1(f)) than those in Group 0. Although not significant, participants in Group III had higher IL-17A levels than those in Group 0 (Figure 1(h)). In contrast, high IL-13 was strongly associated with severe fibrosis (Figure 1(c)).

### 3.3. Cytokine Levels in the Nonfibrotic and Fibrotic Groups According to Sex

**Figure 2:**
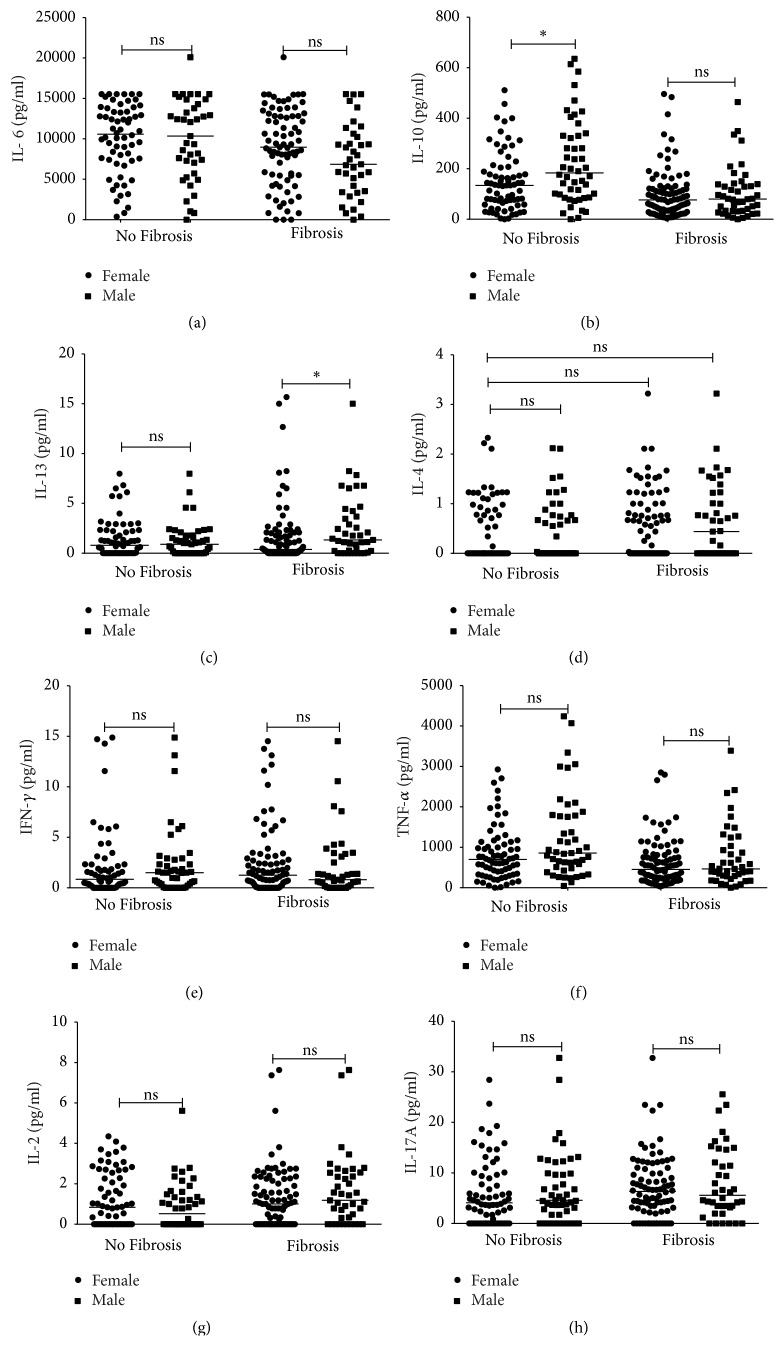
Cytokine Levels in female and male participants with or without fibrosis. Median values are shown by the horizontal line. The Mann–Whitney test was used to compare differences in median cytokine expression in individuals without fibrosis and those with fibrosis. ^*∗*^*p* ≤ 0.05.

To determine whether sex did influence cytokine expression, comparisons were made between female and male participants with and without fibrosis. In this analysis, IL-10 differed significantly between females and males in the nonfibrotic group (Figure 2(b)) whereas IL-13 was high in males with fibrosis compared to females in the same group (Figure 2(c)). No statistically significant differences in the other measured cytokines were observed in the two groups.

## 4. Discussion

In the current study, we have observed that immune responses determined by the expression of Th1, Th2, Th17, and regulatory cytokines in response to* Schistosoma *egg antigen vary in each stage of* S. mansoni* disease. It appears that individuals expressing high levels of IL-6, IL-10, and TNF-*α* tend to be protected against severe hepatosplenic schistosomiasis. On the other hand, high levels of IL-13 correlated with disease severity. Furthermore, we found that the expression of most of these cytokines was not significantly influenced by sex. We further demonstrate for the first time the possible protective role of IL-6 against severe hepatic fibrosis in a population chronically exposed to* S. mansoni* parasites.

Although hepatosplenic schistosomiasis is common in areas endemic for* S. mansoni* parasites, severe disease characterized by extended fibrosis leading to portal vein occlusion and subsequent hypertension, ascites, and formation of oesophageal varices occurs in a subset of individuals with the infection [12, 26]. We have shown in our recent work that, among individuals with* S. mansoni* disease, less than 10% develop severe disease characterized with manifestations mentioned above [23]. The mechanisms surrounding the pathogenesis of* S. mansoni* hepatosplenic disease are centered on a well coordinated network of immune responses involving type 1 response in the early stages of the infection to a more intense type 2 response in the chronic egg deposition stage [5, 27].

Early studies had shown that Th2 associated cytokines (IL-4, IL-5, and IL-13) are the major drivers of pathology in* S. mansoni* disease [4, 5, 28]. In our study, IL-13 was significantly correlated with disease severity confirming earlier reports that this cytokine induces hepatic fibrosis [29, 30]. The current findings are consistent with those from Brazil where a significant association between high IL-13 levels and severe hepatic fibrosis in* S. mansoni* infected individuals was documented [31]. On the contrary, no associations were found between this cytokine and the different stages of fibrosis in hepatosplenic patients with* S. mansoni* infection in another study [32]. Of interest in the present study was the possible protective role of IL-6 against severe fibrosis. IL-6 was highly expressed in individuals with no or mild fibrosis than in those with moderate/severe fibrosis in our study. The lack of IL-6 is associated with increased liver injury and enhanced activity of hepatic stellate cells resulting in fibrosis [33]. Recently, the therapeutic potential of IL-6 was shown by its ability to reverse liver fibrosis [34]. Increased levels in our study participants without fibrosis could suggest that IL-6 modulates* S. mansoni* related immune responses that could otherwise be harmful to the host. However, the actual mechanisms through which this cytokine could be involved in the immunopathogenesis of* S. mansoni* disease remain unclear. As IL-6 is highly pleiotypic, further studies may be needed to correlate it with respective gene activity in order to understand its protective mechanism.

Contrary to other studies that have documented an association between high levels of TNF-*α* with* S. mansoni* disease severity [2, 12, 18], we observed elevated levels of this cytokine in participants with no or mild fibrosis. Our finding is in accordance with previous observations that have shown TNF-*α* to be protective against egg induced liver pathology [16, 35, 36]. In another study, TNF-*α* was only shown to be significantly associated with fibrosis when it was analysed together with IFN-*γ* in a multivariate model [12]. This could mean that the pathogenic role of TNF-*α* is dependent on other cytokines such as IFN-*γ*. In a similar study, the effect of TNF-*α* on fibrosis was age and sex dependent. [2]. We, however, did not find any association between these two variables and TNF-*α* expression in relation to fibrosis.

Although we did not find statistically significant effect of IFN-*γ* on hepatic fibrosis, individuals with severe fibrosis produced less of this cytokine than did those with no fibrosis. IFN-*γ* inhibits the activity of hepatic stellate cells (HSC) which negatively affects the proliferation and synthesis of collagen and other extracellular matrices important for fibrosis [11, 14, 37]. Its lack of association with fibrosis in our study population could be attributed to the short incubation time of the SEA stimulated whole blood. Therefore, longer incubation times should be considered in future studies.

In the current study, low IL-10 levels were associated with moderate/severe hepatic fibrosis while IL-13 was raised in this group. This may indicate that, due to an ineffective regulatory immune response, there is excessive expression of IL-13 and subsequent uncontrolled deposition of extracellular matrix in the hepatic spaces resulting in fibrosis. The regulatory role of IL-10 has been well demonstrated. For instance,* S. mansoni* infected mice deficient in IL-10 show polarized immune responses which are detrimental to the survival of these animals [38, 39]. Similarly, human participants with severe hepatic fibrosis tend to express less IL-10 compared to nonfibrotic individuals [2]. In our study, a linear relationship between IL-10 and the expression of IL-6 and TNF-*α* in participants with mild or no fibrosis was observed. Consistent with the present findings, McGeachy and colleagues showed that IL-10 is upregulated in the presence of IL-6 [40]. It is plausible to suggest that these two cytokines act in synergy to modulate immune responses thus preventing the development of severe hepatic fibrosis.

To further understand whether Th17 CD4^+^-T cells are involved in the pathogenesis of hepatic fibrosis, we investigated the role of IL-17A in relation to* S. mansoni* disease severity. Although a slight increase was observed in the severe hepatic fibrotic group compared to other groups, the difference was not statistically significant. IL-17A is one of the cytokines produced by the Th17 cells [41] and has been implicated in the pathogenesis of a number of disease conditions including the S*chistosoma* associated fibrosis [42].

Despite having well defined groups of hepatic fibrosis, the present study had no* S. mansoni* negative control group. A negative control group could have provided baseline data on the distribution of cytokines in uninfected individuals. In spite of this limitation, we were still able to draw conclusions based on the distinct cytokine profiles observed in the fibrotic groups in comparison to the nonfibrotic group.

## 5. Conclusion

In the present study, we have shown that high levels of IL-6, IL-10, and TNF-*α* could be protective against severe* S. mansoni *hepatic disease. On the other hand, our findings of high IL-13 in the severe fibrotic group are in agreement with previous studies suggesting that this cytokine is involved in fibrosis.

## Figures and Tables

**Table 1:** Characteristics of study participants, by degree of hepatic fibrosis.

Characteristics	Group 0 *n* = 111	Group I *n* = 59	Group II *n* = 43	Group III *n* =31	*p* value
Median age, in yrs (IQR)	28 (16–42)	29 (17–40)	38 (34–46)	39 (28–46)	0.00
*Sex, n (%)*					
Male	44 (50.6)	21 (24.1)	15 (17.2)	7 (8.1)	
Female	67 (42.7)	38 (24.2)	28 (17.8)	24 (15.3)	0.38
^a^*Intensity of infection, n (%)*					
Light	29 (46.0)	17 (27.0)	10 (15.8)	7 (11.1)	
Moderate	16 (50.0)	6 (18.7)	5 (15.6)	5 (15.6)	
Heavy	14 (73.7)	3 (15.8)	2 (10.5)	0 (0.0)	0.43
PVD_ _^b^ , mean ± SEM	10.16 ± 0.23	10.71 ± 0.57	14.32 ± 0.57	17.63 ± 1.01	0.00

The groups were defined and categorized based on liver image pattern as stated in Materials and Methods.  ^a^Intensity of infection is based on 114 *S. mansoni* ova positive participants and this was graded according to the WHO criteria [24].  ^b^PVD is the portal vein diameter.

**Table 2:** Median (interquartile range) cytokine levels (pg/ml) in the nonfibrotic and fibrotic groups.

Cytokine	No fibrosis *n* = 111	Fibrosis *n* = 133	*p* value
IL-2	0.93 (0, 2.28)	0.78 (0, 1.92)	0.365
IL-4	0.20 (0, 1.11)	0 (0.0, 0.88)	0.264
IL-6	11208 (7571, 14714)	8058 (4146, 11030)	<0.001
IL-10	139.6 (76.80, 239.20)	77.7 (32.0, 128.70)	<0.001
IL-13	0.57 (0.17, 2.92)	1.45 (0.26, 4.67)	<0.001
IL-17A	4.59 (1.7, 10.0)	6.64 (3.18, 12.63)	0.05
IFN-*γ*	1.35 (0.17, 2.92)	0.78 (0.23, 2.17)	0.23
TNF-*α*	722.2 (404.8, 1174.6)	451.0 (218.4, 828.2)	<0.001

The Mann–Whitney test was used to compare differences in median cytokine levels in individuals without fibrosis and those with fibrosis.
